# Case report: Stereotactic MR-guided adaptive radiotherapy for inoperable urothelial carcinoma at the renal pelvis

**DOI:** 10.3389/fonc.2023.1284417

**Published:** 2024-01-08

**Authors:** Wajana Thaweerat, Pittaya Dankulchai

**Affiliations:** Division of Radiation Oncology, Department of Radiology, Faculty of Medicine Siriraj Hospital, Mahidol University, Bangkok, Thailand

**Keywords:** adaptive radiotherapy, MR-guided radiotherapy, renal pelvis tumor, stereotactic body radiotherapy, transitional cell carcinoma, urothelial carcinoma

## Abstract

We report the case of an 87-year-old woman with upper tract urothelial carcinoma at the left renal pelvis. She received stereotactic body radiotherapy of 35 Gy in five fractions for palliative treatment of hematuria that was delivered by a 1.5-T magnetic resonance (MR) imaging-guided linear accelerator. Her symptom was relieved after treatment, and posttreatment imaging revealed a complete response of the primary tumor. Thus, this case showed that stereotactic MR-guided radiotherapy could be an appealing option for inoperable patients although radiotherapy is infrequently mentioned in the current treatment guideline of upper tract urothelial carcinoma. Daily adaptive planning from MR images obtained before treatment could improve the target dose and minimize the organ at risk dose. This may lead to a decrease in radiation adverse effects including worsening renal function due to the renal pelvis tumor’s proximity to the kidney.

## Introduction

Upper tract urothelial carcinoma (UTUC) at the renal pelvis and ureter accounts for less than 10% of all urothelial carcinoma cases and renal tumor cases, but the incidence has gradually increased over the past several decades (1). Surgical treatment is a preferred modality in localized nonmetastatic disease, while systemic therapy and immunotherapy are the first-line treatments in the metastatic setting, with surgery offered to selected patients as a palliative treatment (2). Nevertheless, radiotherapy has a very limited role in UTUC as an adjuvant treatment after radical nephroureterectomy (RNU) for locoregional control, which is still unclear due to insufficient data and no other indications are mentioned in the current guideline of the European Association of Urology (EAU) (2).

Radiotherapy has been successfully used in treating urothelial carcinoma at other locations such as locally advanced bladder cancer in bladder preservation therapy as an alternative to radical cystectomy (3) and oligoprogressive or oligorecurrent urothelial carcinoma (4). Thus, radiotherapy could be an option for inoperable UTUC patients or palliative management that was recommended as one of the palliative treatments by the American Urological Association (AUA) (5). Moreover, radiation treatment by stereotactic body radiotherapy at a lesion adjacent to the kidney has a low toxicity rate with an acceptable impact on renal function as demonstrated in studies of renal cell carcinoma treatment (6). Hence, we presented the case of metastatic UTUC at the left renal pelvis treated with stereotactic magnetic resonance (MR)-guided adaptive radiotherapy as a palliative treatment for gross hematuria.

## Case description

An 87-year-old woman has underlying diseases of hypertension, dyslipidemia, and chronic kidney disease stage V. She has a history of UTUC at the right renal pelvis. She underwent right RNU with bladder cuff sparing on November 2019, which the pathological report revealed to be high-grade papillary urothelial carcinoma size 1 cm invading beyond the muscularis propria into the renal parenchyma with clear resection margins. She did not receive any adjuvant treatment. She also has a history of recurrent non–muscle-invasive bladder cancer, which was first diagnosed on January 2021. She received multiple sessions of transurethral resection of bladder tumor (TURBT) in which the pathology report from the last session on March 2022 showed high-grade papillary urothelial carcinoma.

**Figure 1:**
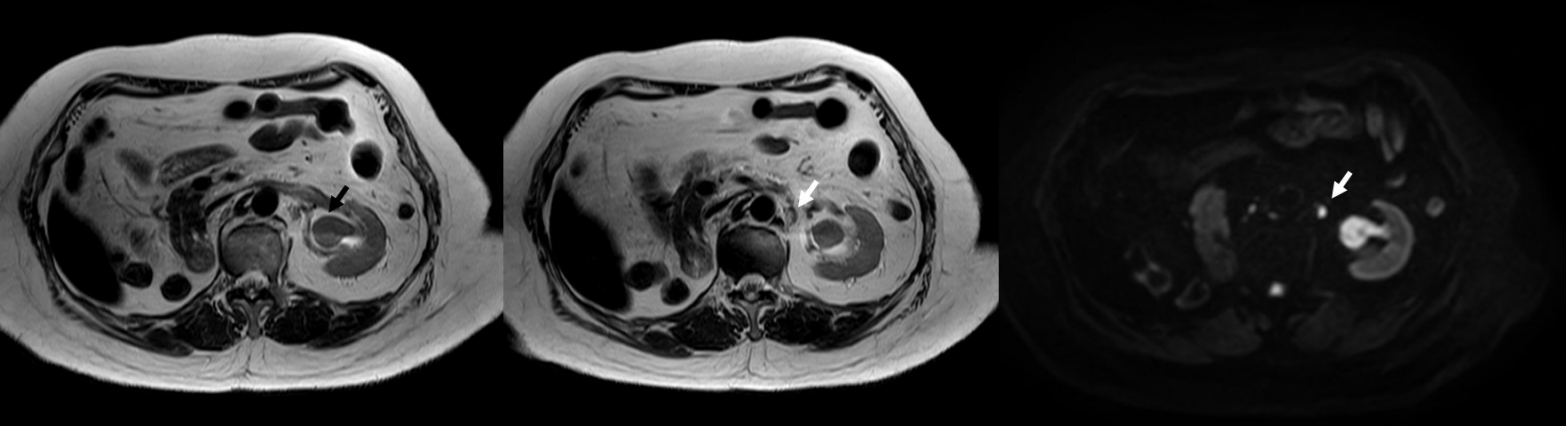
MRI urography demonstrating a lesion at the left renal pelvis size of 2.6 cm × 1.9 cm × 2.9 cm with restricted diffusion but no gross extraluminal extension (black arrow) and a 0.7-cm left para-aortic lymph node with prominent restricted diffusion but preserved hilar fat adjacent to the aforementioned lesion (white arrow).

She visited the urology outpatient clinic for her routine follow-up on March 2022. She complained of gross hematuria for 2 weeks. Office cystoscopy revealed multiple papillary growths at the bladder dome. TURBT was performed on March 2022, and the pathology report showed high-grade noninvasive papillary urothelial carcinoma. Nevertheless, she still had intermittent gross hematuria. MR urography on August 2022 revealed a left renal pelvic mass with a size of 2.6 cm × 1.9 cm × 2.9 cm with lower calyceal extension and a 0.7-cm left para-aortic lymph node with prominent restricted diffusion adjacent to the left pelvic mass (**Figure 1**), but no bladder mass or gross mass at the surgical bed of the right nephrectomy is noted. Further computed tomography (CT) of the chest demonstrated multiple solid pulmonary nodules scattered at both lungs with a size up to 0.9 cm, which is likely pulmonary metastasis. Left ureteroscope that was performed on September 2022 showed a papillary mass at the left renal pelvis without ureteric mass or stone. Ureteroscopic biopsy of the left renal pelvic mass was performed, and the pathology report revealed high-grade papillary urothelial carcinoma without subepithelial tissue invasion.

**Figure 2:**
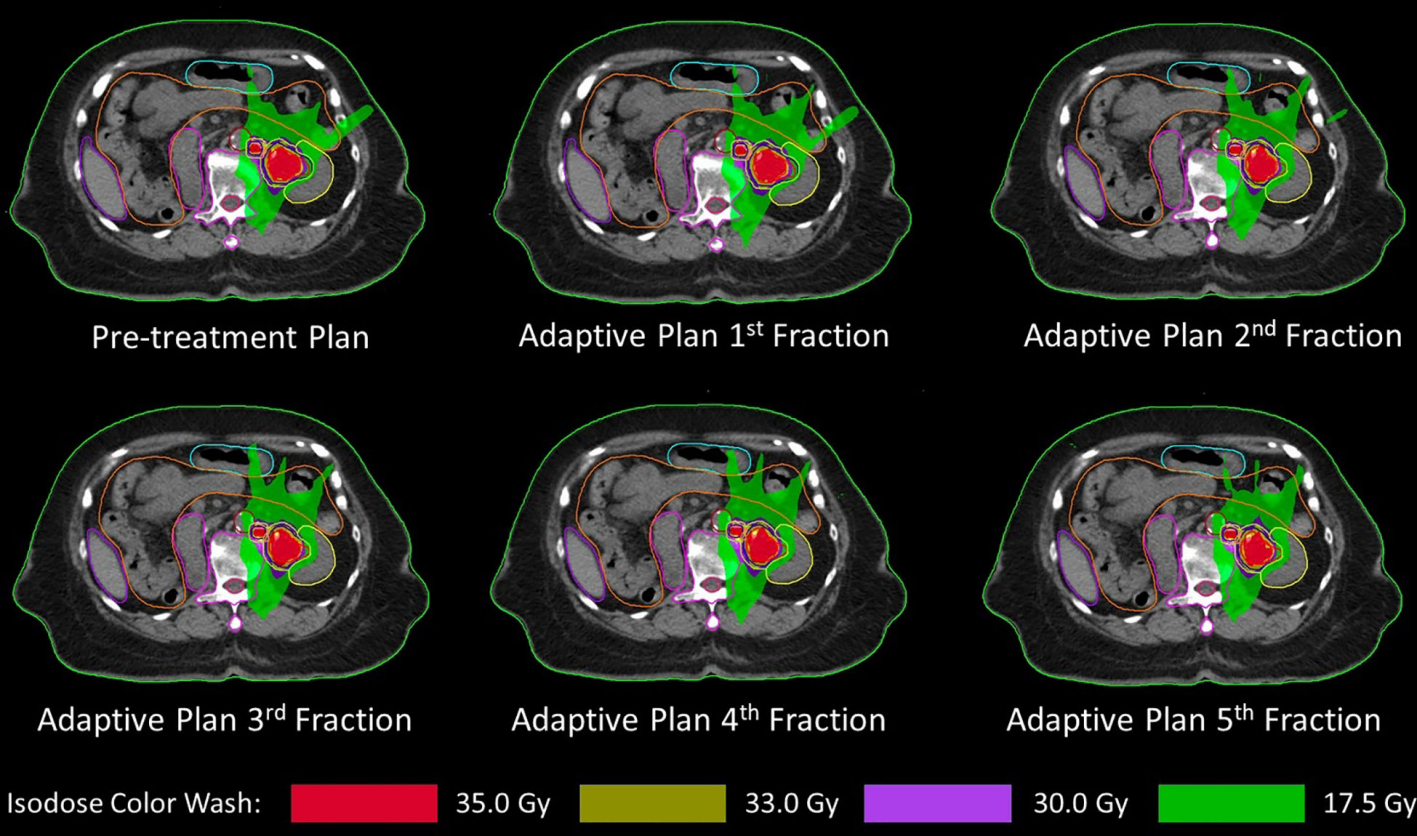
Pretreatment plan and all five daily adaptive plans showing gross tumor volume (red line), clinical target volume (blue line), and planning target volume (light green line) with left kidney (yellow line), spinal cord (dark pink line), duodenum (pink line), and small bowel (orange line).

She was diagnosed with urothelial carcinoma at the left renal pelvis with lung metastasis. Treatment with immunotherapy was discussed by the medical oncologist but was unaffordable by the patient. The patient and her relative opted for the best supportive care. However, she still had gross hematuria while taking oral tranexamic acid. She visited the radiation oncology clinic for consultation on palliative radiotherapy to treat hematuria. Both CT and MR simulation were performed on December 2022. The gross renal pelvic tumor and the adjacent enlarged para-aortic lymph node were delineated as gross tumor volume GTVp and GTVn, respectively. Then, clinical target volume CTVp and CTVn resulted from a 3-mm expansion of GTVp and GTVn excluding renal parenchyma. Lastly, planning target volume (PTV) resulted from a summation of a 3-mm expansion of CTVp and CTVn excluding renal parenchyma (**Figure 2**).

Stereotactic body radiotherapy was planned to deliver 35 Gy in five daily consecutive fractions with a 1.5-T MRI-guided linear accelerator (MR-Linac). Adapt-to-position (ATP) workflow was used in each fraction by rigidly registering the pretreatment CT image with daily MR images. Registered structures will be verified by a radiation oncologist to ensure that targets and organs at risk (OARs) are correctly identified. However, if there is an anatomical change that cannot be corrected by the ATP workflow, the adapt-to-shape (ATS) workflow will be initiated and contours will be edited by the radiation oncologist. The plan will be reoptimized and reevaluated by the radiation oncologist that the target coverage and dose of OARs are acceptable. Tumor motion will be monitored by T2-weighted cine MR images that it was within the target contour before starting the treatment delivery. All five fractions were delivered by using the ATP workflow, and the daily adaptive plan of each fraction was demonstrated in **Figure 2** with the pretreatment plan. The dose of target and OAR are shown in **Table 1**. OAR dose constraint criteria were assessed by following Timmerman’s five-fraction table (7) except kidney that followed the UK consensus on normal tissue dose constraint for stereotactic radiotherapy (8) that has a specific criterion for a solitary kidney. During the treatment period, the patient did not have any additional remarkable symptoms such as nausea or diarrhea.

**Table 1:** Target volume dose coverage and organ at risk dose of pretreatment plan and adaptive plan in each day.

Target volume dose coverage							
Targets	Pretreatment plan	Adaptive plan first fraction	Adaptive plan second fraction		Adaptive plan third fraction	Adaptive plan fourth fraction	Adaptive plan fifth fraction
GTVp V35	86.21%	85.01%	85.77%		86.22%	85.10%	82.00%
GTVn V35	100.00%	100.00%	100.00%		100.00%	100.00%	100.00%
CTVp V33	85.35%	84.65%	85.63%		85.76%	85.39%	83.73%
CTVn V33	99.93%	99.68%	100.00%		100.00%	100.00%	99.86%
PTV V30	92.24%	92.43%	93.05%		92.39%	93.12%	92.12%
Organ at risk dose							
OARs	Constraint	Pretreatment plan	Adaptive plan first fraction	Adaptive plan second fraction	Adaptive plan third fraction	Adaptive plan fourth fraction	Adaptive plan fifth fraction
Left kidney V10	< 45%	37.95%	39.03%	39.46%	39.94%	39.01%	40.24%
Left kidney Dmean	< 10 Gy	9.889 Gy	10.015 Gy	10.152 Gy	10.065 Gy	10.092 Gy	10.235 Gy
Bowel bag Dmax	< 34.5 Gy	27.430 Gy	28.461 Gy	27.677 Gy	28.513 Gy	27.614 Gy	27.874 Gy
Bowel bag V24	< 30 cm^3^	1.423 cm^3^	2.012 cm^3^	2.085 cm^3^	1.368 cm^3^	1.840 cm^3^	1.727 cm^3^
Duodenum Dmax	< 35 Gy	12.356 Gy	12.783 Gy	12.119 Gy	12.869 Gy	12.331 Gy	13.372 Gy
Duodenum V26.5	< 5 cm^3^	0.000 cm^3^	0.000 cm^3^	0.000 cm^3^	0.000 cm^3^	0.000 cm^3^	0.000 cm^3^
Spinal cord Dmax	< 28 Gy	12.356 Gy	15.992 Gy	16.311 Gy	16.868 Gy	15.538 Gy	15.646 Gy
Spinal cord V22	< 0.035 cm^3^	0.000 cm^3^	0.000 cm^3^	0.000 cm^3^	0.000 cm^3^	0.000 cm^3^	0.000 cm^3^

CTVn, nodal clinical target volume; CTVp, primary tumor clinical target volume; Dmax, maximum dose; Dmean, mean dose; GTVn, nodal gross target volume; GTVp, primary tumor gross target volume; PTV, planning target volume; V10, volume receiving 10 Gy; V22, volume receiving 22 Gy; V24, volume receiving 24 Gy; V26.5, volume receiving 26.5 Gy; V30, volume receiving 30 Gy; V33, volume receiving 33 Gy; V35, volume receiving 35 Gy.

**Figure 3:**
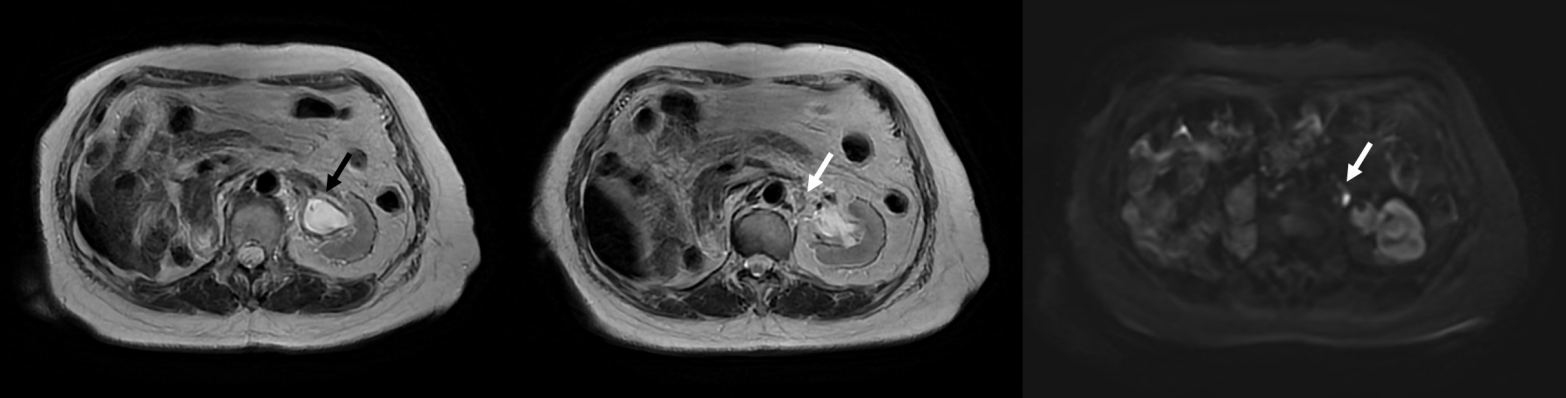
MRI at 3 months posttreatment illustrating the disappearance of a mass at the left renal pelvis with no restricted diffusion (black arrow) and a stable 0.7-cm left para-aortic lymph node with preserved hilar fat (white arrow).

MR imaging of the whole abdomen that was performed 3 months post-radiotherapy demonstrated the disappearance of the left renal pelvic mass without evidence of other gross mass lesions along the left ureter and an unchanged 0.7-cm left para-aortic lymph node with preserved hilar fat (**Figure 3**). Thus, the primary lesion is a clinically complete response to the treatment, while the subcentimeter left para-aortic lymph node was a stable disease or might be benign. The clinical follow-up at 4 months was unremarkable. She did not develop any symptomatic gross hematuria, and her complete blood count and renal function were also stable.

## Discussion

RNU may be offered as palliative care to metastatic UTUC patients with a resectable locally advanced tumor for relieving symptoms (2). However, RNU in the metastatic setting improves survival only in patients who received standard chemotherapy and had only one metastatic site, while the survival benefit disappears in patients with more than one metastatic site (9). Since RNU is an invasive procedure and is limited to patients with resectable disease, inoperable patients due to their medical conditions or unresectable tumor might have no alternative for local treatment to alleviate their symptoms. This case demonstrated that stereotactic body radiotherapy can be a noninvasive option for local treatment as palliative care to relieve gross hematuria. However, radiation therapy was meagerly mentioned in the current UTUC guideline.

The current EAU guideline mentioned the role of radiotherapy in UTUC as an adjuvant treatment, which is still controversial (2). In the latest systematic review and meta-analysis including 20 studies with 6,529 patients, adjuvant radiotherapy usually is given in cases with locally advanced disease, positive lymph nodes, or positive surgical margin, and it could only reduce locoregional recurrence risk with similar survival at 3 years and worsening survival at 5 years (10). However, the effect of radiotherapy on survival should be cautiously interpreted due to a small arm of adjuvant radiotherapy, selection bias with higher-risk patients assigned to the radiotherapy arm and treatment techniques, especially in older studies that treated with a larger and less conformal field of treatment leading to underdosed target volume and increased toxicity (10).

Apart from adjuvant treatment, radiotherapy is also reported as salvage therapy and palliative treatment. In contrast to the EAU guideline, the AUA guideline recommended radiotherapy only as one of the palliative treatment modalities for symptomatic relief in inoperable UTUC patients (5). A large retrospective study of salvage or palliative radiotherapy in recurrent or metastatic UTUC showed that the treatment is feasible and effective especially with intensity-modulated radiotherapy (IMRT) to escalate higher dose or concurrent chemotherapy to improve the radiotherapy response (11). In a small case series, five-fraction stereotactic body radiotherapy with dose varied from 35 to 45 Gy is an effective treatment for palliation of gross hematuria from UTUC at the renal pelvis in patients who were unfavorable surgical candidates but have a moderate decline of renal function without patients who required hemodialysis (12). The reported case series uses cone beam CT (CBCT) for pretreatment position adjustment without implanted fiducial marker or intra-fraction motion tracking (12). It is different from this case in which MR imaging was used for inter-fractional adaptive treatment planning and intra-fractional motion monitoring. Structures including target and OARs will be adjusted to match the daily MR image, and the plan will be reoptimized to be comparable to the pretreatment plan. The margin could be decreased due to the reduction of inter-fractional anatomical change, and it will be mainly accounted for intra-fractional motion of the target, which will be confirmed by cine MR images before treatment delivery.

Delivering stereotactic radiotherapy to the lesion adjacent to the kidney might be a concern for patients with a solitary kidney. Nevertheless, a multi-institutional analysis from the International Radiosurgery Oncology Consortium for Kidney for primary renal cell carcinoma (IROCK) revealed that delivering stereotactic radiotherapy to the renal tumor in a solitary kidney has an acceptable impact on renal function (13). For solitary kidney dose constraint, Timmerman’s dose constraint table (7) provides constraint for kidney in general without specific criterion for patients with a single kidney. In this case, the dose constraint for the solitary kidney from UK consensus on dose constraint for stereotactic radiotherapy was used for evaluation, but the recommended constraint is based on the clinical trial of radiotherapy delivered to lung cancer and biliary tract cancer (8). However, IROCK agreed that there is no evidence-based dose constraint of solitary kidney and recommended that normal renal parenchyma should be cautiously spared as much as possible (14). Hence, daily adaptive planning will allow the optimal plan to reduce the renal dose as low as possible each day while maintaining an adequate dose at the tumor. Moreover, IROCK also reported that small bowel is the dose-limiting organ in most institutions (14). The inter-fractional variation of bowel anatomy may result in a better target dose coverage when the bowel is located in a favorable site farther from the target while ensuring that the bowel dose will not exceed the constraint if it is near the target. The daily adaptive planning will verify that the dose to the anatomically altered bowel in each day is acceptable while the target receives a satisfactory dose.

This case also demonstrates a complete response of the primary tumor at the left renal pelvis. Therefore, radiotherapy might be a prominent alternative for the definitive treatment in surgical-ineligible candidates with localized nonmetastatic disease. A few UTUC case series with different radiotherapy techniques demonstrated radiation therapy is locally effective and well tolerated. A case series of nine patients with UTUC at the renal pelvis and seven patients with UTUC at the ureter who were treated with IMRT of 70 Gy in 35 fractions had at least partial response when evaluated with CT at 1 month and had only one recurrent case without any grade 2 or higher toxicity at a median follow-up time of 30 months (15). A retrospective study of nine renal pelvic UTUC and seven ureteric UTUC treated with stereotactic body radiotherapy varying from 20 to 40 Gy in 5–8 fractions revealed that 68.8% of patients have at least partial response at the primary tumor without renal failure requiring dialysis (16). A report of proton beam therapy of 60–66 Gy (or when assuming relative biological effectiveness of 1.1, 66–72.6 Gy RBE) delivered to three patients with UTUC at the renal pelvis showed grade 1–2 toxicity and had one in-field recurrent case at 36 months posttreatment and one distant metastatic case at 28 months posttreatment (17).

Stereotactic body radiotherapy is another technique that can be utilized in tumors at the renal pelvis as a tumor boost after conventional radiotherapy or as a single definitive treatment. Conventional fractionated radiotherapy of 48.0–52.8 Gy in 20–22 fractions followed by partial stereotactic ablative boost radiotherapy 24.0–30.0 Gy in 3–5 fractions at the gross tumor, which had a total biological equivalent dose of 107.5–108.7 Gy when assuming α/β ratio of 10, was presented in a case series with three patients of UTUC at the renal pelvis (18). Patients treated with this two-phase regimen were alive without disease after 8.6–30.9 months of follow-up and had manageable toxicity without renal function impairment or ureteric stricture (18). Definitive treatment with stereotactic body radiotherapy of 50 Gy in four fractions that was presented in a case report of a medically inoperable UTUC patient at the left renal pelvis had only treatable grade 1 hematuria at 3 months posttreatment without other acute or late toxicity and resulted in complete response at reevaluation at 31 months posttreatment (19). Both reports performed daily pretreatment CBCT to ensure the patient’s position before treatment delivery (18, 19). Thus, stereotactic MR-guided radiotherapy could be a promising treatment for definitive radiotherapy, since it could maximize the target dose while sparing the dose to OARs by daily adaptive planning. However, the translation of dosimetric benefits from adaptive planning into clinically meaningful outcomes still requires further investigation.

In conclusion, this case presented that stereotactic MR-guided radiotherapy of 35 Gy in five fractions delivered by a 1.5-T MR-Linac is a safe and effective palliative treatment for patients with UTUC at the renal pelvis who has hematuria. Although radiation treatment still has a very limited role in UTUC at the renal pelvis, stereotactic MR-guided radiotherapy could be an interesting treatment option for nonsurgical candidates in both curative and palliative settings, as the adaptive planning allows daily reoptimization of the treatment plan after the adjustment of target and OARs, which might result in better disease control and decreased toxicity, especially renal function impairment.

## Data availability statement

The original contributions presented in the study are included in the article/supplementary material. Further inquiries can be directed to the corresponding author.

## Ethics statement

The requirement of ethical approval was waived by the Faculty of Medicine Siriraj Hospital, Mahidol University for the studies involving humans. The studies were conducted in accordance with the local legislation and institutional requirements. The participants provided their written informed consent to participate in this study. Written informed consent was obtained from the patient for the publication of this case report and any accompanying images.

## Author contributions

WT: Conceptualization, Data curation, Investigation, Methodology, Supervision, Writing – original draft, Writing – review & editing. PD: Conceptualization, Data curation, Investigation, Methodology, Supervision, Writing – original draft, Writing – review & editing.
